# Machine learning applications in placenta accreta spectrum disorders

**DOI:** 10.1016/j.eurox.2024.100362

**Published:** 2024-12-24

**Authors:** Mahsa Danaei, Maryam Yeganegi, Sepideh Azizi, Fatemeh Jayervand, Seyedeh Elham Shams, Mohammad Hossein Sharifi, Reza Bahrami, Ali Masoudi, Amirhossein Shahbazi, Amirmasoud Shiri, Heewa Rashnavadi, Kazem Aghili, Hossein Neamatzadeh

**Affiliations:** a Department of Obstetrics and Gynecology, School of Medicine, Iran University of Medical Sciences, Tehran, Iran; b Department of Obstetrics and Gynecology, Iranshahr University of Medical Sciences, Iranshahr, Iran; c Shahid Akbarabadi Clinical Research Development Unit, School of Medicine, Iran University of Medical Sciences, Tehran, Iran; d Department of Pediatrics, Hamadan University of Medical Sciences, Hamadan, Iran; e Department of Cardiology, Hamadan University of Medical Sciences, Hamadan, Iran; f Neonatal Research Center, Shiraz University of Medical Sciences, Shiraz, Iran; g Student Research Committee, School of Medicine, Shahid Sadoughi University of Medical Sciences, Yazd, Iran; h Student Research Committee, School of Medicine, Ilam University of Medical Sciences, Ilam, Iran; i Student Research Committee, School of Medicine, Shiraz University of Medical Sciences, Shiraz, Iran; j Student Research Committee, School of Medicine, Tehran University of Medical Sciences, Tehran, Iran; k Department of Radiology, School of Medicine, Shahid Rahnamoun Hospital, Shahid Sadoughi University of Medical Sciences, Yazd, Iran; l Mother and Newborn Health Research Center, Shahid Sadoughi University of Medical Sciences, Yazd, Iran

**Keywords:** Machine Learning, Radiomics, Placenta Accreta Spectrum, Diagnostic Methods, Surgical Planning, Predictive Models

## Abstract

This review examines the emerging applications of machine learning (ML) and radiomics in the diagnosis and prediction of placenta accreta spectrum (PAS) disorders, addressing a significant challenge in obstetric care. It highlights recent advancements in ML algorithms and radiomic techniques that utilize medical imaging modalities like magnetic resonance imaging (MRI) and ultrasound for effective classification and risk stratification of PAS. The review discusses the efficacy of various deep learning models, such as nnU-Net and DenseNet-PAS, which have demonstrated superior performance over traditional diagnostic methods through high AUC scores. Furthermore, it underscores the importance of integrating quantitative imaging features with clinical data to enhance diagnostic accuracy and optimize surgical planning. The potential of ML to predict surgical morbidity by analyzing demographic and obstetric factors is also explored. Emphasizing the need for standardized methodologies to ensure consistent feature extraction and model performance, this review advocates for the integration of radiomics and ML into clinical workflows, aiming to improve patient outcomes and foster a multidisciplinary approach in high-risk pregnancies. Future research should focus on larger datasets and validation of biomarkers to refine predictive models in obstetric care.

## Introduction

Placenta accreta spectrum disorders (PAS) are characterized by the abnormal invasion of placental villi into the myometrium, with varying severities classified as placenta accreta, increta, and percreta. The rising global cesarean delivery rates have led to an increased incidence of these disorders, significantly elevating the risks during pregnancy and contributing to maternal morbidity and mortality [1], [2]. Prevalence estimates for PAS may reach 67 %, with maternal mortality rates potentially up to 7 %. Complications from undiagnosed PAS include cystotomy and ureteral injury, highlighting the critical need for effective prenatal diagnosis [3], [4]. Magnetic Resonance Imaging (MRI) is a key imaging tool for assessing PAS due to its high sensitivity and specificity in detecting placental adhesion and invasion. International guidelines recommend MRI for high-risk populations, and recent advancements, including diffusion-weighted imaging and radiomics, are further improving the understanding of PAS characteristics and informing clinical decisions [5], [6].

In recent years, machine learning (ML) techniques for predicting PAS have emerged, reflecting a shift toward more advanced diagnostic methods that enhance accuracy and improve patient outcomes [7]. Conditions under PAS, such as placenta accreta, increta, and percreta, involve abnormal placental attachment and myometrial invasion, which pose significant risks, especially during delivery [8], [9]. The increasing incidence of these disorders, predominantly due to higher cesarean section rates, underscores the need for effective predictive models to identify at-risk patients early in pregnancy [10]. Recent studies indicate that combining various imaging modalities and clinical data can improve PAS prediction. Ultrasound remains the primary antenatal diagnostic tool, with a sensitivity of about 89.5 % and a negative predictive value of 98 %, although its positive predictive value is around 68 % [11], [12]. MRI offers deeper insights into placental invasion, achieving sensitivity and specificity rates of 94.6 % and 84.2 %, respectively [7], [13]. By training ML algorithms on datasets that include ultrasound and MRI findings along with clinical factors like myometrial thickness and surgical history, models can enhance diagnostic precision. Some studies propose scoring systems and nomograms that integrate clinical and imaging features to assess PAS likelihood, such as Kamel et al.'s scoring model, which identified significant ultrasound indicators [14]. Additionally, Chen et al. developed a nomogram combining clinical and MRI features for predicting invasive placenta, which could be refined further through ML. ML applications may also allow for personalized management plans for PAS patients by forecasting condition severity and potential complications, helping healthcare providers prepare for surgical interventions more effectively [15]. This proactive strategy aims to reduce maternal morbidity and mortality while addressing the psychological impacts on patients with high-risk pregnancies.

This review article explores recent advancements in machine learning for predicting PAS disorders. It synthesizes current research to highlight the effectiveness of various modeling approaches, including T2-weighted MRI analyses and radiomics, while discussing how deep learning techniques enhance diagnostic accuracy. The article emphasizes the implications of these innovations for clinical practice and patient outcomes, showcasing the transformative potential of machine learning in high-risk obstetric care.

## Classification of placenta accreta spectrum using ML

ML plays a crucial role in classifying and managing the subtypes of the PAS. Table 1 provides a summary of the machine learning applications used for the classification and management of PAS. Wang et al. introduced a deep learning pipeline that uses MRI for automated PAS evaluations. Their study, which involved a large cohort of 540 pregnant women suspected of PAS, employed nnU-Net for placenta segmentation and DenseNet-PAS for diagnosis, achieving area under the curve (AUC) scores of 0.860 and 0.897 for internal and external cohorts, respectively. This method outperformed experienced radiologists and highlighted the importance of prior knowledge about the utero-placental borderline for diagnostics [16]. Verde et al. further evaluated manual segmentation techniques in MRI-derived radiomic analysis for predicting PAS in patients with placenta previa, finding that three-dimensional volumes of interest (VOIs) outperformed two-dimensional regions of interest (ROIs), achieving a mean validation score of 0.761 [17]. Young et al. explored ultrasound texture features combined with ML, developing two models that achieved promising accuracies of 87 % and 92 % for PAS detection, thus enhancing clinical decision-making [18]. Zheng et al. created a cascaded deep semantic-radiomic-clinical (DRC) model focused on T2-weighted MRI, with AUC values of 0.850 and 0.841 in internal and external validations, demonstrating its effectiveness compared to traditional methods [19]. Bartels et al. conducted a bi-centre retrospective analysis using radiomic analysis of T2-weighted MRI to assess PAS severity, revealing variability in sensitivity and specificity and underscoring the need for model validation. Additionally, Bartels et al. validated prior models and introduced a clinical-radiomic model that combined specific radiomic features with clinical variables, achieving an AUC of 0.713, indicating potential for improving prenatal assessments in high-risk pregnancies [20]. Moreover, Leitch et al. demonstrated that MRI radiomic features could predict surgical needs with an accuracy of 0.88 for hysterectomy prediction [21].

**Table 1:** Summary of machine learning applications in classifying and managing PAS.

**Study**	**Methodology**	**Key Findings**
**Wang et al. 2024**	Deep Learning Pipeline using MRI	Achieved AUC scores of 0.860 and 0.897 for internal and external cohorts; outperformed experienced radiologists.
**Verde et al. 2023**	Manual Segmentation Techniques	3D volumes of interest (VOIs) outperformed 2D regions of interest (ROIs) with a mean validation score of 0.761.
**Young et al. 2024**	Ultrasound Texture Features with ML	Developed two models achieving accuracies of 87 % and 92 % for PAS detection.
**Zheng et al. 2024**	Cascaded Deep Semantic-Radiomic-Clinical (DRC) Model	AUC values of 0.850 and 0.841 in internal and external validations.
**Bartels et al. 2023**	Bi-centre Retrospective Analysis	Variability in sensitivity and specificity; AUC of 0.713 with clinical-radiomic model.
**Leitch et al. 2022**	MRI Radiomic Features	Predicted surgical needs with an accuracy of 0.88 for hysterectomy prediction.
**Romeo et al. 2019**	Texture Analysis of MRI	k-NN classifier achieved 98.1 % accuracy; Naïve Bayes performed least at 80.5 %.
**Young et al. 2024**	ML for PAS Detection	Achieved test accuracies of 87 % and 92 % with integrated features based on weighted z-scores.
**Sun et al. 2019**	Intraplacental Texture Features	Gradient boosting classifier achieved 100 % sensitivity and 95.2 % accuracy.
**Zhu et al. 2024**	nnU-Net for PAS Disorders	Training cohort accuracy of 0.771; testing cohort accuracy of 0.825.
**Zheng et al. 2023**	Cascaded Deep DRC Model	AUC of 0.850, combining clinical histories and imaging techniques.
**Ye et al. 2022**	Collaborative Approach	Combined model yielded an AUC of 0.872, enhancing predictive accuracy.
**Peng et al. 2023**	Deep Learning Radiomics	Achieved AUCs exceeding 0.880 across datasets.
**Bartels et al. 2023**	Predictive Modeling for FIGO Grades	Sensitivity of 0.64, specificity of 0.93; advocated for standardized methodologies.
**Yasar et al. 2022**	Proteomic Data with ML	XGBoost model achieved accuracy of 0.962; KDR and AMH identified as potential biomarkers.
**Futterman et al. 2024**	Multicenter Study on Surgical Morbidity	AUC of 0.79; identified key predictors for surgical outcomes.
**Shazly et al. 2021**	Validation of PAR-A Score	AUC of 0.85 for predicting massive blood loss; strong clinical utility.

Footnote: This table summarizes recent studies utilizing machine learning techniques in the classification and management of Placenta Accreta Spectrum (PAS), highlighting methodologies and key findings that contribute to improved diagnostic accuracy and clinical decision-making.

## Texture analysis of ultrasound and MRI in ML

Texture analysis has emerged as a powerful technique in medical imaging, particularly in the evaluation of placental abnormality, such as the PAS. Utilizing both ultrasound and MRI, studies have highlighted significant advancements in diagnostic accuracy through the application of ML algorithms to extracted texture features. In a study by Romeo et al., ML techniques were applied to analyze MRI-derived texture features for predicting PAS in patients with placenta previa, using pre-operative MRI scans from 64 patients—20 of whom were confirmed positive for PAS (comprising 12 accreta, 7 increta, and 1 percreta). The study utilized multiple rounded regions of interest (ROIs) on T2-weighted images to extract texture analysis features, implementing a rigorous methodology that included the Synthetic Minority Over-sampling Technique for dataset balancing and a 75/25 % train-test split. Among various algorithms tested, the k-nearest neighbors classifier achieved the highest accuracy of 98.1 %, with 98.7 % precision, 97.5 % sensitivity, and 98.7 % specificity using only 26 features, while the Naïve Bayes algorithm showed the least performance at 80.5 % accuracy [7]. In 2024, Dylan Young et al. utilized ML for PAS detection through two models: one employing texture features from ultrasound scans and the other using a linear classifier with integrated features based on weighted z-scores. Their approach, combining classical ML and statistical methods for feature selection, resulted in superior performance, achieving test accuracies of 87 % and 92 %, and 5-fold cross-validation accuracies of 88.7 (4.4) and 83.0 (5.0), respectively. These models demonstrate effective, non-invasive diagnostic tools for improved PAS detection, aiding clinicians in reducing unnecessary interventions and facilitating earlier management planning for delivery. Furthermore, findings indicate a strong correlation between gestational age and texture features in diffusion-weighted imaging, with substantial differences in pixel intensity analysis between PAS and normal placentas. Radiomics-based ML models utilizing MRI texture data have shown the potential to predict PAS with high sensitivity (100.0 %) and specificity (88.5 %), outperforming traditional visual assessments by radiologists [18]. Overall, these non-invasive diagnostic tools promise to aid clinicians in reducing unnecessary interventions and facilitating earlier management planning for delivery.

## Radiomics-based prediction models for placenta accreta spectrum

Radiomics-based prediction involves extracting numerous quantitative features from medical imaging data using advanced computational techniques to create predictive models for patient outcomes, disease progression, or treatment responses. This approach utilizes high-dimensional imaging data, encompassing shape, texture, and intensity information. Recent advancements in ML and radiomics have significantly enhanced the predictive capabilities for PAS disorders, as evidenced by multiple research studies focusing on T2-weighted MRI. Each study contributes unique insights into the application of various imaging features, model strategies, and clinical data to better identify and manage this complex condition. In a noteworthy study by Sun et al., intraplacental texture features derived from conventional placental MRI were employed to predict invasive placentation. The analysis involved 99 women with confirmed placental invasion and 56 with simple placenta previa, utilizing MRI sequences post-24 weeks of gestation. The segmenting method applied to sagittal turbo spin echo (TSE) and balanced turbo field echo (bTFE) images enabled the extraction of textural features which were subsequently analyzed by a gradient boosting classifier. Impressively, the classifier achieved a sensitivity of 100 %, specificity of 88.5 %, and accuracy of 95.2 %, underscoring the potential for advanced texture analysis in clinical scenarios involving suspicious placental conditions [22]. Building upon this foundation, researchers like Hao Zhu et al. developed a computerized diagnostic model specifically targeting PAS disorders, which incorporated manual outlining of preoperative T2-weighted imaging. Their model utilized a nnU-Net network for automatic segmentation, followed by the extraction of relevant radiomic features that formed the basis of their predictive algorithm. The results indicated robust performance metrics, with a training cohort accuracy of 0.771 and a testing cohort accuracy of 0.825. In external validation, their model also demonstrated high sensitivity, highlighting its diagnostic relevance [23]. Likewise, Bartels et al. focused on the validation of a multivariate radiomic model using T2-weighted MRI data over several years. Their study showed significant improvements over previous models, achieving an AUC of 0.713 through the incorporation of both radiomic features and clinical history. The new model's performance metrics underscored the role of ML in evolving clinical practices for managing high-risk births involving PAS [24]. Furthermore, Zheng et al. introduced a cascaded deep DRC model that surpassed conventional diagnostic models, attaining an AUC of 0.850 and demonstrating strong performance in external testing cohorts. This innovative approach combined clinical histories like prior uterine surgeries with advanced imaging techniques, providing significant support in surgical planning for patients [19]. In a similar vein, Ye et al. employed a collaborative approach that integrated clinical data, radiomics, and deep learning, evaluating a significant number of pregnant women across multiple centers. Their combined model yielded an AUC of 0.872, suggesting that a multidisciplinary methodology can substantially bolster predictive accuracy [25]. Leitch et al. drew attention to the utility of MRI through extensive radiomic feature extraction to predict the necessity for hysterectomy and diagnose PAS. Their analysis involved a diverse cohort demonstrating promising accuracies of 0.88 and 0.92 in various classification tasks. This work not only affirms the potential of radiomic techniques to aid clinical decision-making but also stresses the need for precise imaging protocols tailored to the complexities of PAS [21]. Complementing this, Peng et al. ventured into deep learning radiomics, formulating a model that displayed superior diagnostic performance compared to traditional methods. They achieved AUCs exceeding 0.880 across different datasets, reflecting the robustness of integrating advanced deep learning techniques with conventional clinical evaluations [26]. Verde et al. contributed significantly to the discourse by investigating various manual segmentation methodologies for MRI-derived radiomic analysis. Their findings reinforced the critical nature of segmentation techniques on predictive accuracy for PAS, highlighting that specific focus areas, particularly the retroplacental myometrium, provided the best performance metrics. Overall, these studies collectively support the notion that leveraging ML frameworks, robust imaging protocols, and integrated clinical datasets can dramatically enhance the identification and management of PAS disorders. They reinforce the importance of adopting advanced analytical techniques in high-risk obstetric scenarios, paving the way for improved outcomes through informed clinical decision-making. The evolving landscape of radiomics and ML in obstetric imaging stands as a testament to the power of interdisciplinary collaboration aimed at enhancing maternal and fetal health [17]. Collectively, the findings from these studies illustrate the transformative potential of radiomics in enhancing diagnostic accuracy and clinical decision-making in managing complex placental disorders like PAS, paving the way for improved outcomes in high-risk pregnancies.

## Radiomics-based prediction of FIGO grading for PAS severity

Radiomics, which entails the extraction of quantitative features from medical imaging, is increasingly recognized as a promising method for predicting the severity of PAS disorders, especially in classifying FIGO grades. A recent study by Bartels et al. examined data from 41 women diagnosed with PAS, specifically targeting the predictive value of radiomic features from MRI scans for severe FIGO grade 3 cases. The researchers implemented four multivariate predictive modeling techniques: LASSO, Random Forest (RF), k-Nearest Neighbors (kNN), and Support Vector Machine (SVM). Results from the study indicated a sensitivity of 0.64, specificity of 0.93 in the univariate analysis, an accuracy of 0.58, and an AUC of 0.77, suggesting promising initial findings. In contrast, the SVM model exhibited lower sensitivity (0.30) but retained similar accuracy (0.58), indicating variability in model performance across different approaches. The authors advocated for a standardized methodology for feature extraction and image segmentation to mitigate inconsistencies noted in previous research. Calibration curves showed satisfactory correspondence between predicted probabilities and observed rates of FIGO grade 3 PAS, demonstrating that the models reliably estimated the likelihood of PAS for both FIGO grade 3 and non-grade 3 cases. All models performed comparably in predicting FIGO grade 3 PAS, achieving ROC values above 50 % and specificity exceeding 60 %. Notably, RF outperformed in terms of accuracy in the superior region of interest (ROI) compared to the inferior ROI, while kNN displayed greater specificity in the inferior region [24]. Overall, despite the dataset's limitations, the findings highlight the potential of radiomics to shape individualized care strategies for women with severe PAS and emphasize the necessity for standardized methodologies to enhance predictive capabilities in obstetrics. Future research should focus on larger datasets and the incorporation of multi-modal data, including clinical histories and genomic information, to further refine prediction accuracy.

## AI versus radiologists in diagnosing PAS

Recent studies show that AI is increasingly proficient in diagnosing PAS disorders and often outperforms experienced radiologists. ML models have demonstrated high accuracy in identifying PAS, with an AUC of 0.849 compared to 0.744 for radiologists, indicating a superior ability for discrimination in diagnosis. One model utilizing T2-weighted MRI images achieved an accuracy of 82.5 %, with a sensitivity of 83.0 % and specificity of 82.2 %, while radiologists reached only 75.3 % accuracy, a sensitivity of 53.2 %, and a specificity of 85.0 % [23], [27], [28]. This highlights the ML model's greater sensitivity, which is crucial for the accurate identification of PAS. Incorporating clinical and radiomics data further enhances diagnostic performance, evidenced by a combined model achieving an AUC of 0.833. Various ML algorithms, including XGBoost and Adaboost, have recorded accuracy rates exceeding 95 %, demonstrating their effectiveness in detecting PAS [23]. Although radiologists possess valuable skills, their performance can be inconsistent, especially in complex cases with subtle signs of PAS. AI acts as a complementary tool, aiding radiologists in making more accurate diagnoses and reducing their workload, which allows them to concentrate on more intricate cases [29]. Furthermore, AI can enhance the confidence of less experienced radiologists by offering standardized support, particularly in environments with limited access to specialized training. Overall, integrating AI into clinical workflows promises to improve diagnostic accuracy and efficiency, ultimately leading to better patient outcomes for individuals with PAS.

## Integration of ML with biomarkers

ML has emerged as a powerful tool for enhancing the diagnostic accuracy and prognostic evaluation of clinical outcomes related to PAS disorders by integrating biomarkers. A significant study conducted by Yasar et al. in 2022 utilized proteomic data from 26 women, both with and without PAS, to develop ML models using XGBoost and Adaboost, following variable selection via the Lasso method and a 5-fold cross-validation technique. The XGBoost model demonstrated superior performance, achieving an impressive accuracy of 0.962, along with a balanced accuracy of 0.950, perfect sensitivity of 1.00, and a specificity of 0.90. These results affirm the model's efficacy in classifying PAS, particularly highlighting KDR (Vascular Endothelial Growth Factor Receptor 2) and AMH (Anti-Müllerian Hormone) proteins as potential biomarkers for diagnosis and monitoring. The study demonstrates that ML, particularly algorithms like XGBoost, can enhance the accuracy of PAS diagnosis using proteomic data. If validated, these biomarkers could improve timely diagnosis and monitoring of PAS, benefiting patient outcomes and informing treatment strategies [30]. Further research is necessary to confirm the reliability and generalizability of these biomarkers in diverse populations. Integrating ML with biomarker data can enable personalized risk assessments and improve management protocols, leading to better follow-up and surgical planning. As research advances, these methods are expected to become standard in obstetric care, enhancing outcomes and reducing PAS-related complications.

## Prediction of surgical morbidity using ML

The use of ML models to assess surgical morbidity in PAS disorders marks a significant advancement in maternal healthcare. Recent studies show that ML can effectively identify key demographic and obstetric factors impacting surgical outcomes, thus improving risk stratification and clinical decision-making. High AUC values of these predictive models demonstrate their potential in preoperative planning and resource optimization. Enhanced scoring systems offer insights into severe maternal morbidity risks, underscoring the need to integrate ML into obstetric practice. For example, a multicenter study by Futterman et al. analyzed 401 confirmed PAS cases, identifying 213 variables related to demographics, obstetrical history, and prenatal imaging. The model successfully predicted surgical morbidity with an area under the receiver operating characteristic curve (AUC) of 0.79 and demonstrated a positive predictive value (PPV) of 0.79 and a negative predictive value (NPV) of 0.76. Key predictors included the completion of hysterectomy, body mass index (BMI), and timing of delivery [31]. Shazly et al. further validated the placenta accreta risk-antepartum (PAR-A) score in 86 women, revealing an AUC of 0.85 for predicting massive blood loss and 0.88 for intensive care unit admissions, suggesting its clinical utility [32]. A separate study by Shazly et al. in 2021, involving 727 women, reported AUC values of 0.84–0.90 for various clinical outcomes, highlighting factors like parity and placental site as important predictors [33]. Additionally, Leonard et al. developed a comorbidity scoring system using data from over 919,000 births in California, identifying severe maternal morbidity rates and emphasizing PAS as the highest risk comorbidity, with an adjusted risk ratio of 30.5. The scoring system exhibited strong performance metrics, enabling improved risk assessment and management strategies [34]. Overall, these advancements represent a pivotal shift toward integrating ML in clinical practice, improving predictive accuracy and facilitating individualized risk assessments for enhanced patient care in managing PAS disorders.

## Discussion

The integration of ML into the management of the PAS marks a transformative advancement in prenatal care, significantly enhancing diagnostic accuracy and clinical decision-making. Recent studies reveal that deep learning methodologies outperform traditional assessments, including those conducted by experienced radiologists. Wang et al. utilized MRI along with nnU-Net for placenta segmentation and DenseNet-PAS for diagnosis, achieving impressive AUC scores of 0.860 and 0.897 across diverse cohorts, thus confirming the efficacy of automated systems [16]. In a complementary study, Verde et al. demonstrated that three-dimensional vo VOIs yielded superior results compared to two-dimensional ROIs in radiomic analysis, emphasizing the necessity for advanced imaging techniques in conjunction with robust ML approaches [17]. Young et al. further explored ultrasound texture features integrated with ML, resulting in high detection accuracies that enrich the diagnostic arsenal for high-risk pregnancies [18]. Zheng et al. introduced a cascaded deep DRC model that effectively merges radiomic and clinical data to improve PAS diagnostics [19]. Bartels et al. pointed out the variability in model sensitivity and specificity, stressing the importance of cross-validation in radiomic studies. Moreover, Leitch et al. showcased the predictive power of MRI radiomic features in anticipating surgical interventions, achieving 0.88 accuracy for hysterectomy predictions [21].

Radiomics-based prediction has notably advanced obstetric imaging, particularly in managing PAS. By extracting quantitative features from medical imaging, particularly MRI, this method allows for detailed analysis of tissue characteristics that aids clinical decision-making. Research indicates the effectiveness of ML techniques in predicting patient outcomes, disease progression, and treatment responses. A notable study by Sun et al. highlighted the use of intraplacental texture features to predict invasive placentation, achieving 100 % sensitivity and 88.5 % specificity, thus enhancing diagnostic accuracy in high-risk pregnancies [22]. Additionally, Zheng et al. emphasized a comprehensive approach by combining clinical histories with radiomic features, demonstrating improved diagnostic accuracy and valuable support for surgical planning. However, variations in model performance across studies call for standardized methods in feature extraction and image segmentation to enhance reliability and generalizability. The evolution of ML techniques, such as nnU-Net for automated segmentation, suggests more precise analyses [19]. Collaborative efforts, like those in Ye et al., can utilize larger datasets for robust analyses, but challenges persist, including limited sample sizes and variability in imaging protocols. Future research should focus on large-scale multicentric studies and incorporate diverse data types, such as genomic information, to refine predictive accuracy and deepen understanding of PAS. Overall, advancements in radiomics and ML signify a transformative shift in PAS diagnosis and management. By employing advanced analytical techniques and comprehensive patient data, healthcare providers can improve individualized treatment strategies, enhancing outcomes for mothers and infants alike. The ongoing development of radiomics-based predictive models has the potential to redefine clinical practices and improve care for women facing the complexities of PAS [25].

Integrating ML into diagnostics and prognostics for PAS disorders is becoming increasingly vital in maternal healthcare. Research shows that AI systems using advanced ML algorithms outperform traditional radiological assessments in terms of accuracy, sensitivity, and specificity, with reported AUCs effectively discriminating between PAS and non-PAS cases. ML models achieve high accuracy rates, particularly with T2-weighted MRI images, reaching up to 82.5 %, while the combination of clinical and radiomic data further enhances diagnostic performance. This multifactorial approach is crucial for PAS, a complex condition that often presents subtle signs easily overlooked by human assessors. Furthermore, identifying specific biomarkers like KDR and AMH through proteomic analyses presents promising avenues to enhance PAS diagnosis, enabling personalized risk assessments and tailored management strategies. ML also aids in predicting surgical morbidity, enabling clinicians to stratify patients based on demographic and obstetric factors influencing outcomes, with AUCs for predicting complications between 0.79 and 0.88. Moreover, incorporating AI and ML into clinical workflows supports experienced radiologists and assists in training less experienced practitioners through standardized assessments for complex decision-making. Despite positive results from various studies, challenges related to validation, generalizability, and clinical acceptance remain. Continued research and broader studies involving diverse populations are essential to confirm the efficacy and reliability of these models. Ultimately, integrating ML into diagnosing and managing PAS disorders signifies a transformative shift in obstetric care, enhancing diagnostic accuracy, optimizing surgical planning, and refining risk assessments to significantly improve maternal and fetal outcomes. As these models become part of routine clinical practice, the future of managing PAS looks promising, with potential to reduce complications associated with this challenging condition.

## Clinical implications

Integrating ML into the diagnostic and management processes for PAS has transformative clinical implications for maternal healthcare. By enhancing diagnostic accuracy through advanced imaging and predictive analytics, ML models can facilitate early PAS detection, enabling timely interventions to reduce complications for both mother and infant. ML's capability to analyze complex datasets, including imaging features and clinical histories, allows for personalized risk assessments and informed decision-making in high-risk pregnancies. Additionally, ML’s predictive power in forecasting surgical morbidity can help clinicians more effectively stratify patients, optimizing surgical planning and resource allocation. As ML systems improve the training of less experienced practitioners and provide standardized assessments, they can bridge knowledge gaps and enhance overall clinical competency in managing PAS. However, widespread adoption will necessitate overcoming challenges related to model validation and acceptance in clinical settings, emphasizing the need for ongoing collaboration and research to ensure these advancements translate into improved patient outcomes.

## Challenges and limitations

Despite significant progress in integrating ML into the diagnosis and management of PAS, several challenges remain. A primary concern is the validation and generalizability of ML models across diverse patient populations and clinical settings, which can affect prediction reliability. Variability in imaging protocols and sample sizes complicates method standardization, leading to inconsistent study performance. There is also a need for rigorous cross-validation to ensure model robustness for real-world applications. Furthermore, the acceptance of AI and ML technologies among healthcare practitioners poses a challenge, as reliance on automated systems may raise concerns about diminishing clinical expertise and the necessity for adequate training. Integrating ML into clinical workflows also requires addressing logistical and infrastructural barriers, such as data privacy and system interoperability. Tackling these challenges is essential for the successful implementation and adoption of ML applications in obstetric care to effectively enhance maternal and fetal outcomes.

## Future directions

Future advancements in ML techniques could significantly enhance the diagnosis and management of PAS disorders through methodological innovations, emerging technologies, collaboration, and ethical considerations. Key future research areas include algorithm optimization, focusing on refining existing ML algorithms and exploring new ones to improve predictive accuracy while reducing overfitting, employing methods like ensemble learning and transfer learning. The incorporation of multimodal data, such as ultrasound, MRI, and clinical parameters, can yield more robust models, as evidenced by successful integrations of radiomics features with clinical data. Moreover, developing ML models for real-time data processing could streamline clinical workflows by providing immediate diagnostic insights for radiologists during imaging procedures. Emerging technologies, particularly AI and big data, can enhance ML capabilities by automating data preprocessing and leveraging large, diverse datasets to improve model generalizability. Advanced imaging techniques, including 3D ultrasound and functional MRI, may also offer additional insights for ML analysis, leading to improved diagnostic outcomes. Collaboration between researchers and clinicians is vital for creating practical ML models, necessitating interdisciplinary partnerships and multicenter trials to validate efficacy across various clinical environments. Additionally, educating clinicians on interpreting ML outputs will be crucial for effective tool utilization. Addressing ethical implications, such as data privacy, bias in training datasets, and ensuring informed consent for patient data usage, is also essential [35], [36], [37]. By confronting these challenges, the future of ML in diagnosing PAS disorders holds significant promise for enhancing diagnostic accuracy and patient outcomes.

## Conclusion

The integration of ML and radiomics into the classification and management of PAS has markedly improved diagnostic accuracy and clinical decision-making. Studies demonstrate the effectiveness of various ML algorithms and imaging techniques, especially MRI and ultrasound texture analysis, in enhancing PAS identification. Advanced methods like deep learning and detailed radiomic feature extraction outperform traditional diagnostics, enabling earlier and more precise evaluations in high-risk pregnancies. Collaborative research highlights the importance of robust imaging protocols and effective segmentation methods to optimize these models' performance. Interdisciplinary approaches that combine clinical expertise with ML can significantly improve PAS management, leading to better prenatal assessments and outcomes for mothers and infants. The ongoing validation and refinement of predictive models are crucial for ensuring their reliability in clinical environments. Furthermore, ML has shown enhanced sensitivity in detecting PAS, proving to be a valuable tool for radiologists. These models can also predict surgical morbidity related to PAS, aiding in preoperative planning. Standardized research methodologies are needed to refine predictive capabilities and enhance clinical practices. Future research should focus on validating these technologies within diverse populations, paving the way for more personalized and efficient care for women affected by PAS.

## CRediT authorship contribution statement

**Amirhossein Shahbazi:** Writing – original draft. **Ali Masoudi:** Methodology. **Reza Bahrami:** Investigation. **Mohammad Hossein Sharifi:** Data curation. **Seyedeh Elham Shams:** Data curation. **Fatemeh Jayervand:** Conceptualization. **Sepideh Azizi:** Conceptualization. **Hossein Neamatzadeh:** Conceptualization, Validation. **Mahsa Danaei:** Conceptualization. **Kazem Aghili:** Validation. **Maryam Yeganegi:** Conceptualization, Investigation. **Heewa Rashnavadi:** Investigation. **Amirmasoud Shiri:** Writing – review & editing.

## Ethics approval

This article does not include any studies involving human participants or animals conducted by the authors.

## Funding

The authors stated that no funding was associated with this work.

## Declaration of Competing Interest

The authors declare that they have no known competing financial interests or personal relationships that could have appeared to influence the work reported in this paper.

## Data Availability

Datasets generated or analyzed during this study are available from the corresponding author upon reasonable request.
